# The Combined Treatment with Ketogenic Diet and Metformin Slows Tumor Growth in Two Mouse Models of Triple Negative Breast Cancer

**DOI:** 10.21203/rs.3.rs-3664129/v1

**Published:** 2023-12-20

**Authors:** Karen Schmidt, Amber Thatcher, Albert Grobe, Linda Hicks, Haiwei Gu, Dorothy D. Sears, Lesley G. Ellies, Leonid Kalachev, Eugene Kroll

**Affiliations:** University of Montana Division of Biological Sciences; University of Montana Division of Biological Sciences; Silverlake Research Corporation; University of Montana Division of Biological Sciences; Arizona State University School of Life Sciences; Arizona State University School of Life Sciences; University of California San Diego; University of Montana; University of Montana Missoula: University of Montana

**Keywords:** hypoxic tumor, glycolytic tumor, systemic glucose limitation, ketogenic diet, metformin

## Abstract

**BACKGROUND:**

Many tumors contain hypoxic microenvironments caused by inefficient tumor vascularization. Hypoxic tumors have been shown to resist conventional cancer therapies. Hypoxic cancer cells rely on glucose to meet their energetic and anabolic needs to fuel uncontrolled proliferation and metastasis. This glucose dependency is linked to a metabolic shift in response to hypoxic conditions.

**METHODS:**

To leverage the glucose dependency of hypoxic tumor cells, we assessed the effects of a controlled reduction in systemic glucose by combining dietary carbohydrate restriction, using a ketogenic diet, with gluconeogenesis inhibition, using metformin, on two mouse models of triple-negative breast cancer (TNBC).

**RESULTS:**

We confirmed that MET − 1 breast cancer cells require abnormally high glucose concentrations to survive in a hypoxic environment in vitro. Then, we showed that, compared to a ketogenic diet or metformin alone, animals treated with the combination regimen showed significantly lower tumor burden, higher tumor latency and slower tumor growth. As a result, lowering systemic glucose by this combined dietary and pharmacologic approach improved overall survival in our mouse model by 31 days, which is approximately equivalent to 3 human years.

**CONCLUSION:**

This is the first preclinical study to demonstrate that reducing systemic glucose by combining a ketogenic diet and metformin significantly inhibits tumor proliferation and increases overall survival. Our findings suggest a possible treatment for a broad range of hypoxic and glycolytic tumor types, one that can also augment existing treatment options to improve patient outcomes.

## Introduction

Aggressively growing tumors develop hypoxic microenvironments due to insufficient and haphazard tumor vascularization (1–3). Chronic tumor hypoxia promotes metastasis (4, 5), increases angiogenesis (6, 7), inhibits the immune response (8, 9) and interferes with apoptosis (10). Furthermore, tumor-derived micrometastases are initially avascular and, therefore, exist in a state of acute hypoxia (11–13). The hypoxic status of a tumor also correlates with resistance to chemo-, radio- and immunotherapies, advanced stages of malignancy and poor clinical prognosis (10, 14, 15).

Cancer cells readily adapt to hypoxic conditions via activation of hypoxia-inducible factors (16–18). Downstream signaling promotes the overexpression of hexose transporters (19, 20) and the eventual depolarization of mitochondrial inner membranes, which inhibits oxidative phosphorylation (OXPHOS) (21). This effect, first noted by Louis Pasteur (22), forces hypoxic cells to rely on oxygen-independent glycolysis for their energetic and anabolic needs (13, 23, 24). Some cancer cell types evolve to retain the glycolytic phenotype even in the presence of oxygen, as noted by Otto Warburg (25).

Provided enough glucose is available, hypoxic tumor cells rapidly produce ATP despite the inefficiency of glycolysis compared to OXPHOS (26). Additionally, the increased glycolytic flux may provide ample feedstocks for cellular components (24). This reliance of hypoxic tumor cells on high glucose flux is a *metabolic vulnerability* and offers new strategies and targets for cancer therapies.

Taking advantage of the relative inefficiency of glycolysis, we postulate that a reduction in systemic glucose may check the growth of hypoxic tumors and their metastases while sparing normal tissue. Properly vascularized and oxygenated tissues can catabolize other nutrients such as fatty acids, ketone bodies, glutamine and lactate, all of which require OXPHOS to produce ATP (27). Certain tissues, such as the brain and erythrocytes, predominantly use glycolysis but are able to switch to ketone bodies upon glucose shortage (28, 29) or survive in a mildly hypoglycemic environment (30). This is supported by the fact that mild hypoglycemia (> 60 mg/dL) is well-tolerated in mice (See Results) and is not considered life-threatening in humans (31).

To control systemic glucose, all possible sources of carbohydrates must be addressed. Exogenous (dietary) sources can be controlled with low-carbohydrate (ketogenic) diets, and endogenous glucose production can be partially inhibited by metformin, an antidiabetic agent. Clinically relevant doses of metformin reduce endogenous glucose output by suppressing gluconeogenesis via mitochondrial glycerophosphate dehydrogenase (mGPD) with a resultant change in the redox state of the cytoplasm (32) and indirectly activating starvation signaling (33). Individually, ketogenic diets and metformin are well-tolerated in humans (34) and their anticancer properties, used separately, have been relatively marginal (35–40).

To test whether lowering systemic glucose could affect hypoxic tumors, we applied the combination regimen of a ketogenic diet and metformin to two mouse models of triple-negative breast cancer (TNBC). TNBC often metastasize which is ultimately responsible for more than 90% of breast cancer deaths (41). As TNBC is genetically heterogeneous, effective therapies are lacking (42). TNBC breast tumors are also frequently hypoxic and glycolytic (2), making this type of breast cancer a suitable model to study the effects of reducing systemic glucose.

In this work we evaluate the sensitivity of breast cancer cell lines to glucose levels and describe the effect of inducing mild hypoglycemia in two mouse models of TNBC by analyzing tumor latency, tumor growth rate and overall survival.

## 1. Methods

### Tissue culture.

MET-1 cells (mouse MMTV-PyMT breast cancer cell line (43)) were seeded in T-25 flasks at 30–50% confluence in complete DMEM (4.5% glucose, 10% FBS) and allowed to reach confluence with one medium change. The medium was replaced with complete DMEM containing 0, 0.5, 1.0 or 4.5g/L glucose and duplicate flasks were placed at 37°C in sealed containers with a Gaspak EZ (Beckton Dickinson) and anaerobic indicator strips to confirm the lack of oxygen. As a control, a duplicate set of cultures were grown in the presence of oxygen.

### Animals.

The use of experimental animals followed guidelines in the National Institutes of Health *Guide for the Care and Use of Laboratory Animals*. The experimental protocol was approved by the University of Montana Institutional Animal Care and Use Committee and work was conducted in an AAALAC-certified facility. Forty (40) female FVB mice and 20 female PyMT transgenic mice (B6.FVB/N-Tg(MMTV-PyMT)634Mul/LellJ) were used in this study (Jackson Laboratories, Bangor, ME).

### Tumor cell injection.

Forty (40) FVB mice were anesthetized with 5% isoflurane until recumbent and unresponsive to a toe pinch. Anesthetized animals were placed in a supine position and injected with 0.5×10^6^ Met-1 cells in 2 mg/mL Matrigel (total volume = 50 μL) into L4 and R4 mammary pads using a 25-gauge needle.

### Diet and metformin dosing.

FVB and PyMT transgenic mice were randomized into four groups: 1) C group – control group maintained on a standard mouse chow diet (Teklad 2020x), 2) M group – standard chow plus metformin, 3) K group - Ketogenic diet (Teklad TD.96355) and 4) KM group - Ketogenic diet plus metformin. Diet and water were available ad *libitum*. Animals in the M and KM groups were given metformin in drinking water at 5 g/L supplemented with 2 g/L Stevia for palatability. Water consumption was measured every two days, and the concentration of metformin was adjusted accordingly.

### Metformin level in mouse blood.

Plasma samples were mixed with methanol and centrifuged. Supernatants were vacuum dried and reconstituted in 40% PBS/60% acetonitrile. Quality-Control (QC) sample was pooled from all available samples. External calibration solutions were used to determine the absolute concentrations of metformin. LC-MS/MS was performed on an Agilent 1290 UPLC-6495 QQQ-MS (Santa Clara, CA) system in hydrophilic interaction chromatography (HILIC) mode on a Waters XBridge BEH Amide column. The mobile phase was composed of Solvents A (10 mM ammonium acetate, 10 mM ammonium hydroxide in 95% H_2_O/5% acetonitrile) and B (10 mM ammonium acetate, 10 mM ammonium hydroxide in 95% acetonitrile/5% H_2_O), and the auto-sampler temperature was kept at 4°C The mass spectrometer was equipped with an electrospray ionization source. Targeted data acquisition was performed in multiple-reaction-monitoring mode. The whole LC-MS/MS system was controlled by Agilent Masshunter Workstation software (Santa Clara, CA). The extracted MRM peaks were integrated using Agilent MassHunter Quantitative Data Analysis.

### Vital signs and tumor volume measurements.

Mouse activity was observed/scored daily according to the Murine Behavior Ethogram, with blood glucose and body weight recorded at least weekly for each mouse. Tumor size was best represented by volume, which we selected as the indicator for tumor burden. To calculate volumes, two orthogonal diameters were measured with calipers with an estimated precision of 6.4% (See Supplemental Materials). Each tumor was evaluated by palpation in the third dimension (height) as flat, ovoid or round. Depending on the shape of the tumor, one of the following formulae were used to calculate volume: $"\text{Flat}"\frac{\pi •x•\left(\frac{y^{2}}{4}\right)}{6}$, $"\text{Ovoid}"\frac{\pi •x•y^{2}}{6}$,or $"\text{Round}"\frac{\pi •x•y•\left(\frac{x+y}{2}\right)}{6}$, where *x* is the largest diameter and *y* is the smallest.

### Modeling tumor growth.

An exponential tumor growth model (44) was fit to the data for all treatment groups (C, M, K and KM - *see* Diet and metformin dosing) with the assumption that tumors proliferate at a constant rate for a particular treatment group, while estimated tumor volumes were specific to each mouse. The following exponential model was used:

$$x_{gi}\left(t_{j}\right)=x_{gi}(T)•exp\left(k_{g}•\left(t_{j}-T\right)\right),$$

Where$x_{gi}\left(t_{j}\right)$ was the total tumor volume of the *i*-th mouse from each treatment group (*g* = C, M, K and KM) measured at a time point *t*_*j*_. These volumes were estimated during model fitting. Parameter *k*_*g*_ (1/day) is the tumor growth rate constant for each group. MATLAB nlinfit.m (v. R2018a) was used to fit model equations to data to estimate growth rate constants *k*_*g*_ and “initial” tumor volumes for each animal *x*_*gi*_(*T*). Standard errors for estimated parameters and statistically reliable inferences about tumor growth rates were obtained using the Delta method (linearization) (45) under an assumption of normality.

### Tumor oxygenation levels.

An OxyLite monitor (Optronix, Oxford, UK) was used to measure tumor tissue oxygenation by detecting molecular oxygen in tissues based upon quenching of light emitted by a fluorescent dye, where the quenching is proportional to the pO_2_ and temperature of the surrounding tissue. Animals were anesthetized with 5% isoflurane in oxygen and maintained at 1–2% isoflurane throughout the procedure. Once animals were unresponsive to a toe pinch, a 22-gauge angiocath was inserted into the tumor lengthwise and the needle was removed. The probe was then inserted into the angiocath to the desired position and the angiocath removed while holding the probe in place. The probe was maintained in the desired position for 3 min for the reading to stabilize, the reading recorded, and the probe retracted an additional 3 mm. This procedure was repeated to obtain three or four measurements in tumor tissue (depending on tumor size). Similar measurements of nearby subcutaneous tissues were taken as controls. Ambient air was also measured and recorded for comparison.

## 2. Results

### Low glucose kills hypoxic, but not normoxic, cancer cells in culture

Based on the causal relationship between hypoxia and glucose dependency, we expected that reducing available glucose would adversely affect the growth or viability of hypoxic cancer cells. To test this, we first replicated the oxygen-starved, glucose-dependent tumor microenvironment *in vitro*. Conventional tissue culture conditions offer a hyperoxygenated and hyperglycemic environment, which is far from what tumor cells experience *in situ*. Cell lines are traditionally grown at a much higher oxygen partial pressure, ~ 150mmHg in the atmosphere vs. ~50 mmHg in normal tissue and can be much lower in tumor tissue (46). Moreover, most culture media contain 4.5 g/L glucose vs. ~1 g/L glucose in the blood and even less in cancer tissues (1).

Instead of the conventional tissue culture conditions, we incubated a mouse breast cancer cell line, MET-1 (47), in a hypoxic chamber with different concentrations of glucose in the DMEM medium (0, 0.5, 1.0 and 4.5 g/L), either in the normal (aerobic flasks) or a low (hypoxic flasks) oxygen atmosphere. After 19 h, aerobic flasks showed no indications of decreased cell viability at all glucose concentrations, as evidenced by medium color and 100% cell adherence. In contrast, hypoxic flasks with glucose concentrations of 0, 0.5 and 1.0 g/L displayed a deep pink color with all cells detached from the flask, suggesting cell death. However, the hypoxic ask with 4.5 g/L glucose appeared yellow, indicating partial acidification, with no detached cells. To test the viability of detached cells in these flasks, we attempted to rescue the cells by adjusting the medium to 4.5% glucose and incubating them in the presence of oxygen for an additional 8 h. Detached cells failed to reattach or grow, indicating that they were non-viable. These results show that, under hypoxic conditions, MET-1 cells require abnormally high glucose concentrations to survive and that lowering glucose levels leads to cell death.

#### MET-1 mouse breast cancer tumors are hypoxic.

**Figure 1:**
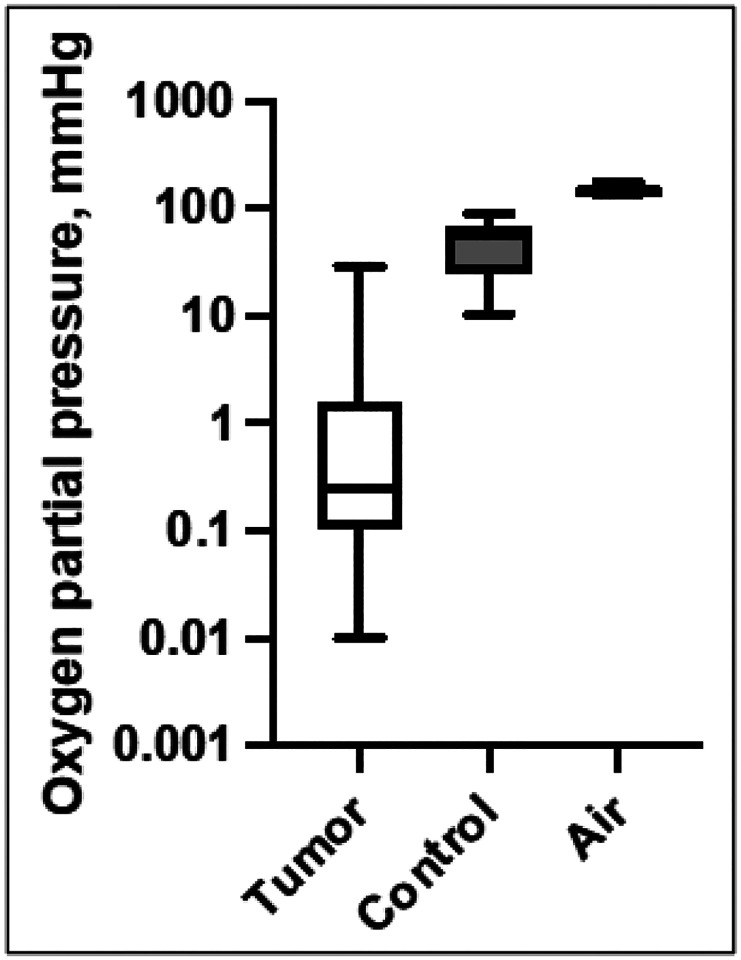
PyMT orthotopic injection tumors display a very low median oxygenation level compared to control (muscle tissue in the vicinity of the tumor). Boxplots depict partial oxygen pressure in respective tissues. The middle line is the median, boxes span the interquartile range, whiskers show the full range of values. To allow for better visualization of the tumor oxygenation range of tumors, the Y axis is logarithmic.

To assess the oxygenation state of tumors in a mouse model, we measured the oxygen partial pressure (pO_2_) in six developed breast tumors after orthotopic injection in FVB mice and compared it to normal tissue. The median partial oxygen pressure in the tumor tissues (pO_2_) was 0.25 mmHg (n = 40, Interquartile range (IQR) 0.10–1.25), while the median pO_2_ for subcutaneous tissue (control) was 57.0 mmHg (n = 13, IQR = 25.4–65.8). The pO_2_ of the surrounding air was 155 mmHg (n = 11, IQR = 139–156) (Fig. 1). While several tumor tissue measurements were as high as in normal tissue, the median pO_2_ was significantly lower (p < 0.0001, Mann-Whitney). Consistent with previous studies (1, 48), these data show that the median tumor tissue oxygenation level in our breast cancer mouse model is approximately one order of magnitude lower than in normal tissue.

### A ketogenic diet-metformin combination regimen delays tumor development

Having confirmed that hypoxic cancer cells require high glucose availability *in vitro* and that tumors in our TNBC mouse model are indeed hypoxic, we next investigated tumor vulnerability to moderately decreased systemic glucose. To this end, we compared the tumor growth effects of combined ketogenic diet plus metformin treatment (KM), ketogenic diet alone (K), metformin treatment alone (M), or control (C) in two *in vivo* models of triple-negative breast cancer.

Animals receiving metformin displayed serum metformin concentrations comparable to previous determinations (49), ranging from 14.8 to 21.8 μM, which approximates human metformin serum concentration at a clinically relevant 1.5g/70kg dose (50).

Mean blood glucose (BG) levels decreased significantly only in the combination ketogenic diet and metformin (KM) group. For FVB animals, the average BG level in the KM group was 123 ± 6 mg/dL, vs. the average for all other groups at 148 ± 3 mg/dL. For PyMT transgenic animals, the average BG level in the KM group was 117 ± 6 mg/dL *vs*. the average for all other groups at 150 ± 11 mg/dL. The lowest BG value (in the KM group) reached 67.2 mg/dL without an apparent change in animal behavior, as scored using the Murine Behavior Ethogram.

We first estimated tumor burden and growth rates in female PyMT transgenic mice that develop random, human-like, hyperplastic mammary adenocarcinomas with lung metastases within the first three months of life (51). The total tumor burden (sum of tumor volumes per animal) was not significant between the control (C), metformin-only (M) and ketogenic diet-only (K) groups. In contrast, the mean tumor burden in the ketogenic diet plus metformin group (KM) was 33.4 ± 3.4% of the mean tumor burden in all other groups throughout the experiment (30 measurements). This is a conservative estimate because animals from control groups with large tumors or large overall tumor burden were euthanized earlier, artificially decreasing the tumor burden ratio. To address this and to make firm statistical inferences, we assessed tumor accumulation using an exponential growth model (*See* Methods).

**Figure 2:**
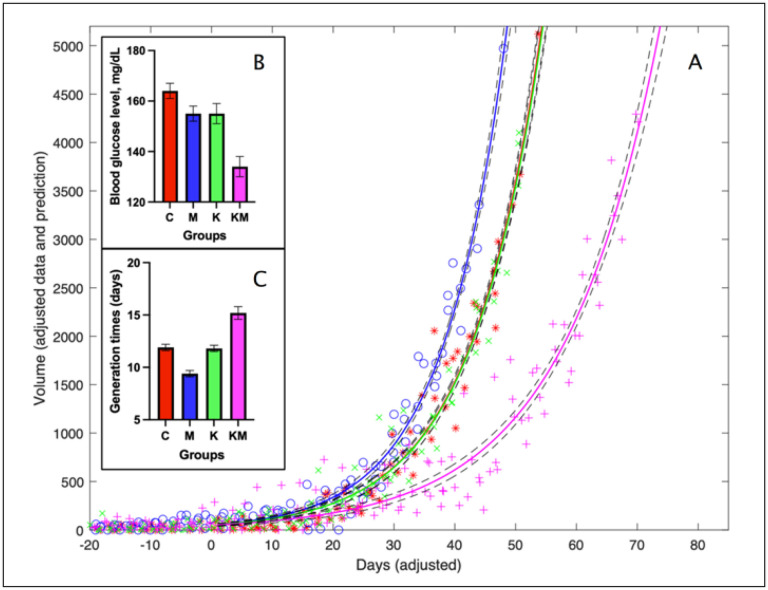
Tumor burden increases at a slower rate in the ketogenic diet/metformin group than in other groups. Red – control (C), blue – metformin only (M), green – ketogenic diet only (K), pink – ketogenic diet plus metformin (KM). A: Time series model fitted curves depict cumulative tumor volumes for groups C (*), M (o), K (x) and KM (+) (mm^3^). Due to the inherent randomness of tumor initiation in this mouse model, we have assigned day “0” for each animal to be equal to a cumulative tumor volume of 10 mm^3^. This makes apparent the difference in the growth rate constant values. Dashed lines indicate 95% confidence bands. B: Differences in blood glucose levels between groups. C: Differences in generation times between groups.

After fitting model parameters to the data, we estimated tumor generation times (the inverse of growth rate constants): C group, 11.9 ± 0.3 days, M group, 9.4 ± 0.3 days, K group, 11.8 ± 0.3 days and KM group, 15.2 ± 0.6 days. Pairwise differences in tumor generation times for the KM group vs. any other group were significantly different (p-values <10^−7^ [z-test]). The combined ketogenic diet plus metformin regimen significantly delayed tumor development compared to other groups (Fig. 2 **and Suppl. Figure 1**).

### Survival is extended on the ketogenic diet-metformin regimen

**Figure 3:**
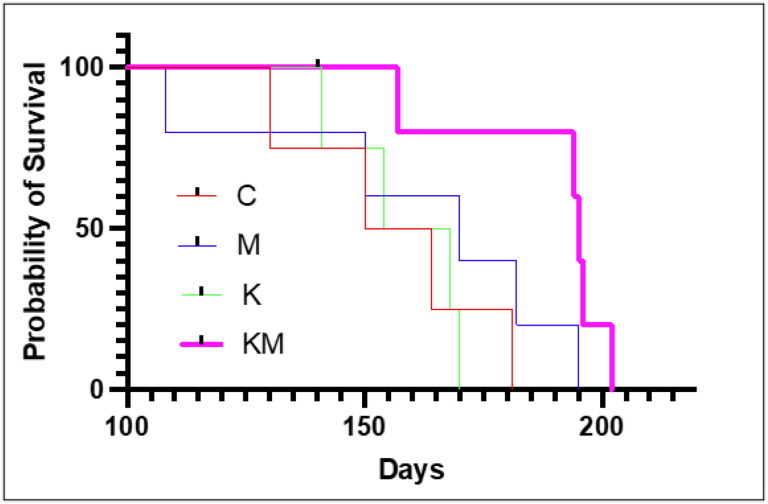
Animals on ketogenic diet and metformin survive longer than animals in other groups. Red – control (C), blue – metformin only (M), green – ketogenic diet only (K), pink – ketogenic diet plus metformin (KM).

Second, we estimated overall survival in female PyMT transgenic mice. Median survival time for each animal from its birthdate to the time it had developed a cumulative tumor mass of 20% of its body weight were: C group − 157 days, M group − 170 days, K group − 161 days and KM group − 195 days. The difference in survival times between KM and the other groups was statistically significant (*p*-valueof 6.89×10^−5^ *χ*^2^ = 15.84, log-tank test) (Fig. 3).

#### Tumor latency is extended on the ketogenic diet-metformin regimen in an orthotopic injection model

Third, we estimated tumor latency, i.e., the period during which the tumor remains undetected. We operationally define tumor latency as the number of days for individual tumors to reach a detectable volume of 100 mm^3^. To synchronize the onset of tumors, we orthotopically injected MET-1 breast cancer cells (bearing the same PyMT construct in their genome as the PyMT transgenic animals) into the L4 and R4 mammary glands of naive FVB mice. Once tumors became detectable, we recorded their dimensions, converted them to volumes, fit the exponential model parameters to these data (see Methods) and then estimated the time it took tumor volumes to reach the detectable level of 100 mm^3^.

**Figure 4:**
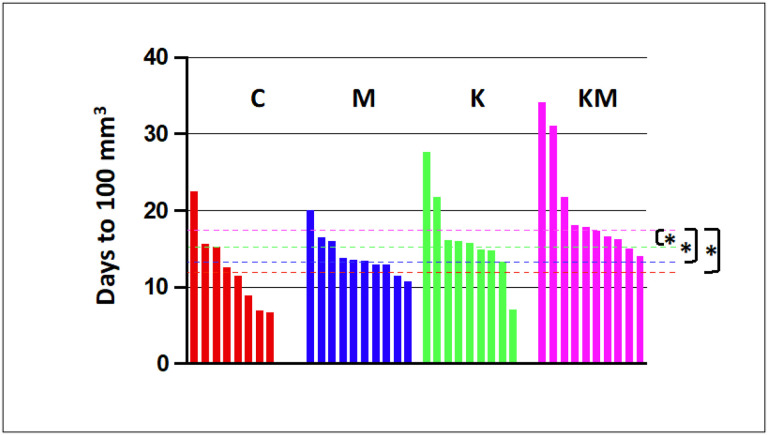
A. Tumor latency (time to reach a detectable 100 mm^3^ total tumor volume) is longer in the KM group compared to the other groups. Each vertical line represents data from a separate mouse. Red – control (C), blue – metformin only (M), green – ketogenic diet only (K), pink – ketogenic diet plus metformin (KM). Dashed lines represent median values for each group. Asterisks denote statistically significant differences at 0.05 significance level (*p*-values are in the text).

The median tumor latency was significantly longer for the KM group animals than other groups (KM vs. C, p = 0.006; KM vs. M, p = 0.002; KM vs. K, *p* = 0.04, one-tailed Wilcoxon rank sum test). These data confirm that the ketogenic diet plus metformin group exhibited a significantly prolonged latency in tumor growth compared to other groups (Fig. 4)

## 3. Discussion

Aggressive tumor proliferation leads to insufficient tumor vascularization, resulting in chronic tumor hypoxia, which forces cancer cells to become highly glycolytic. Here, we show that lowering systemic glucose by the simultaneous reduction in dietary carbohydrates and inhibiting gluconeogenesis delays the development of hypoxic breast cancer *in vitro* and *in vivo*.

The results of this study demonstrate that hypoxic tumor tissues are susceptible to even mild glucose limitation. First, we confirmed that breast cancer cells rely on an abnormally high glucose level to survive in a hypoxic environment in tissue culture. Second, using two aggressive breast cancer mouse models, we showed that a glucose-lowering regimen consisting of a combination of two modalities -- a low carbohydrate (ketogenic) diet and metformin -- significantly decreased tumor burden by 2/3 compared to the control or each modality alone. Moreover, tumors in the ketogenic diet-metformin group grew 38% more slowly, resulting in an additional 31 days of overall survival. This life extension equates to more than three human years (52), a significant increase over the current median TNBC survival of 18 months (42). Third, we showed that the median latency of breast tumors in mice using our combination treatment increased by 36% compared to the median latency of other groups. Lastly, since micrometastases are hypoxic due to the lack of newly–formed vascularization (5, 11), we obtained preliminary evidence that metastasis to the lungs may also be delayed (see Supplementary materials).

Limiting glucose with a combination of a ketogenic diet plus metformin regimen to slow cancer growth has been independently proposed (53, 54) and this combination regimen has been safely used in humans for a different purpose (55). Furthermore, timed metformin dosing during transient hypoglycemia caused by intermittent fasting, strongly inhibited melanoma-derived tumor growth (56). Other ways to limit systemic glucose levels are also under investigation. Several studies described the direct cytotoxic action of metformin in low glucose conditions (57, 58). Additionally, glycolytic tumors have been targeted through inhibition of glycolysis (59), the PI3 Kinase/Akt/mTORc growth signaling pathway (60) or by blocking glucose transport (61, 62). However, as with conventional chemotherapies, tumor evolution can circumvent these targeted chemotherapies, leading to cancer recurrence. Additionally, these molecular approaches may be ineffective or toxic, as some molecular targets are redundant or indiscriminate and normal cells may also rely on these activities. In contrast, lowering systemic glucose via the combined regimen proposed here adopts an “organismic” view of cancer (63) by safely modifying organismal physiology rather than targeting a unique cancer activity.

Confirming our findings, diabetic cancer patients taking metformin exhibit a significantly lower incidence of hepatic, colorectal, mammary and pancreatic cancers and increased survival from colorectal, pulmonary and prostate cancers than those on other antidiabetic medications that do not inhibit gluconeogenesis (66, 67). Most probable explanation is that diabetic patients tend to control their carbohydrate intake better than the general population (68), boosting metformin’s anticancer effect. It follows that a low carbohydrate ketogenic diet in combination with metformin may potentiate metformin’s anti-carcinogenic action in cancer patients regardless of their diabetic status, as we observed in our mouse models.

An alternative explanation is that а concurrent decrease in insulin levels caused by low glucose slows tumor growth. This would mean that in the presence of insulin, the normoglycemic and hypoxic environment should allow cancer cells to proliferate. However, our work shows that the normoglycemic (1g/L) insulin–containing growth medium did not support hypoxic PyMT cancer cell viability. Instead, to survive, MET-1 breast cancer cells required a “diabetic” 4.5g/L glucose level in the DMEM culture medium containing insulin. This observation implies a direct effect of glucose levels on cancer cell growth rather than the indirect effect of lower insulin. While insulin is important in the promotional stage of breast tumorigenesis, a large proportion of advanced ER-negative breast adenocarcinomas do not show a mitogenic response upon insulin signaling in culture (69). Moreover, hyperinsulinemia tends to be irrelevant to breast cancer risk for premenopausal women while potentially increasing it for post-menopausal women (70). Evidence in cell culture, mice and humans demonstrates that hyperglycemia is a *bona fide* risk factor, at least for ER-negative breast cancer such as TNBC.

While we observed a significant decrease in tumor burden, growth rate and an increase in tumor latency with a mild decrease in systemic glucose using a combination of a clinically relevant dose of metformin and a ketogenic diet, the treatment did not inhibit tumor growth altogether. One explanation is that properly oxygenated, and, therefore, nonglycolytic tumor cells would not be susceptible to this regimen. Since well-oxygenated, proliferating cancer cells can be targeted by chemo-, radio- and immunotherapies, our metabolic regimen is a natural candidate for combination with these therapies for synergistic therapeutic effects. Finally, this metabolic regimen may be similarly effective against a broad range of other FDG-PET- positive (glycolytic) tumors in other organs (10, 18, 71).

## Data Availability

All data and materials will be made available provided agreements are in place
